# Characterisation, whole‐genome sequencing and phylogenetic analysis of three H3N2 avian influenza viruses isolated from domestic ducks at live poultry markets of Iran, 2017: First report

**DOI:** 10.1002/vms3.819

**Published:** 2022-06-02

**Authors:** Alireza Abtin, Abdelhamid Shoushtari, Mohammad Hossein Fallah Mehrabadi, Aidin Molouki, Seyed Ali Pourbakhsh, Hadi Pourtaghi, Fatemeh Eshratabadi

**Affiliations:** ^1^ Department of Avian Diseases Research and Diagnostics Razi Vaccine and Serum Research Institute Agricultural Research, Education and Extension Organization (AREEO) Karaj Iran; ^2^ Department of Microbiology Karaj Branch Islamic Azad University Karaj Iran

**Keywords:** avian influenza virus, domestic ducks, H3 subtype, Iran, live poultry market

## Abstract

**Background:**

Avian influenza type A viruses (AIV) can infect a broad range of hosts including human and birds, making them an important viral pathogen with zoonotic potential. Ducks are a known reservoir for many avian viruses including the AIV.

**Objectives:**

To sequence the entire genome of duck‐derived H3N2 and ran comprehensive phylogenetic analysis on them to study their origin.

**Methods:**

In this study, 962 cloacal swabs were collected from domestic ducks at several live poultry markets (LPMs) of Gilan, Mazandaran and Golestan provinces of Iran in the year 2017.

**Results:**

Preliminary assays such as haemagglutination inhibition assay (HI), Neuraminidase Inhibition assay(NI) and RT‐qPCR suggested that 0.5% of the birds were infected by H3 low pathogenic influenza viruses (LPAI). Three isolates were selected for whole genome sequencing. The cleavage site of the HA genes showed a PEKQTR/GLF motif, an indicator of LPAI. Furthermore, BLAST and phylogenetic analyses of the HA gene showed high homology to the Eurasian lineage of H3N8 AIV (95.5%–97.1% to several European and East Asian isolates). However, the NA genes showed high homology (at most 96.5–96.9%) to those belonging to AIV N2 subtype. Furthermore, internal genes showed high homology (96%–98%) to a variety of duck‐origin subtypes and glycoprotein combinations, which were different for each segment. This showed a complex reassortment between different subtypes.

**Discussion:**

This report is the first whole genome sequencing and complete characterisation of H3N2 AIV from Iran.

**Conclusion:**

Such surveillance should continue to study the evolution and possible emergence of viruses with pandemic potential.

## Graphical abstract

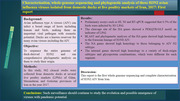

## 1 INTRODUCTION

Influenza viruses are important pathogens that belong to the family Orthomyxoviridae (Swayne, 2020). They are divided into four genera of A, B, C and D that infect vertebrates. Type A influenza viruses that infect birds are generally known as avian influenza viruses (AIV). AIV are of high economic importance as they greatly affect production and breeding of poultry worldwide, as well as having zoonotic potential (Yoon et al., 2014).

The genome of influenza A viruses consist of an eight‐segmented negative‐sense RNA, which is wrapped within a lipid bilayer membrane (Swayne, 2020). The segments are numbered in order of decreasing length. The haemagglutinin (HA, or segment 4) and neuraminidase (NA, or segment 6) genes code for two antigenic glycoproteins on the surface of the lipid membrane. Other segments, namely PB1, PB2, PA, NP, M and NS, code for other structural and internal proteins required for transcription and replication. Currently, influenza A viruses are classified into 18 HA (H1–H18) and eleven NA (N1–N11) subtypes based on the antigenic differences of the HA and NA proteins (Spalding, 2009). Many combinations of these proteins are possible. The reassortment potential of influenza viruses makes them able of exchanging genes with other subtypes. Theoretically, reassortment between two influenza viruses that differ in all eight segments can give rise to 256 distinct genotypes (Phipps et al., 2017).

AIV have the ability to cross the species barrier to infect mammals, and therefore, they have been of special research importance. This ability of AIV was first come under spotlight when it was discovered that the influenza virus causing the 1968 pandemic, better known as Hong Kong flu that killed an estimated 1–4 million people, was in fact derived from an AIV of H3 subtype most similar to those isolated from ducks in Asia (Bean et al., 1992). Over the years, the H3 subtype has become one of the most epidemic subtypes of influenza viruses. Currently, H3 has a wide host range including wild and domestic birds, equine, swine, canine, feline and human (Allen & Ross, 2018; Bean et al., 1992). Moreover, the predominant strain of seasonal influenza in humans since the mid‐2000s has been H3N2 (Allen & Ross, 2018).

On the other hand, waterfowls are natural hosts of influenza A viruses and can infect a variety of domestic birds, which in turn can infect humans (Li et al., 2016; Yoon et al., 2014). Ducks are known waterfowls that are within the family Anatidae of the order Anseriformes. Domestic ducks are highly mobile and they are an interface between wild birds, other domestic birds and mammals (Deng et al., 2013). These birds often live in ponds where many different wild birds visit.

Live poultry markets (LPMs) provide a great environment for interspecies transmission of different diseases. In the current study, cloacal samples from domestic ducks that were being sold at LPMs of the northern provinces of Iran in 2017 were investigated for the presence of avian viruses. From these samples, three H3N2 AIV were isolated, all of which were genetically characterised. In this study, and for the first time in Iran, we have completely sequenced the entire genome of duck‐derived H3N2 and ran comprehensive phylogenetic analysis on them to study their origin.

## 2 MATERIAL AND METHODS

### 2.1 Sample collection

**FIGURE 1:**
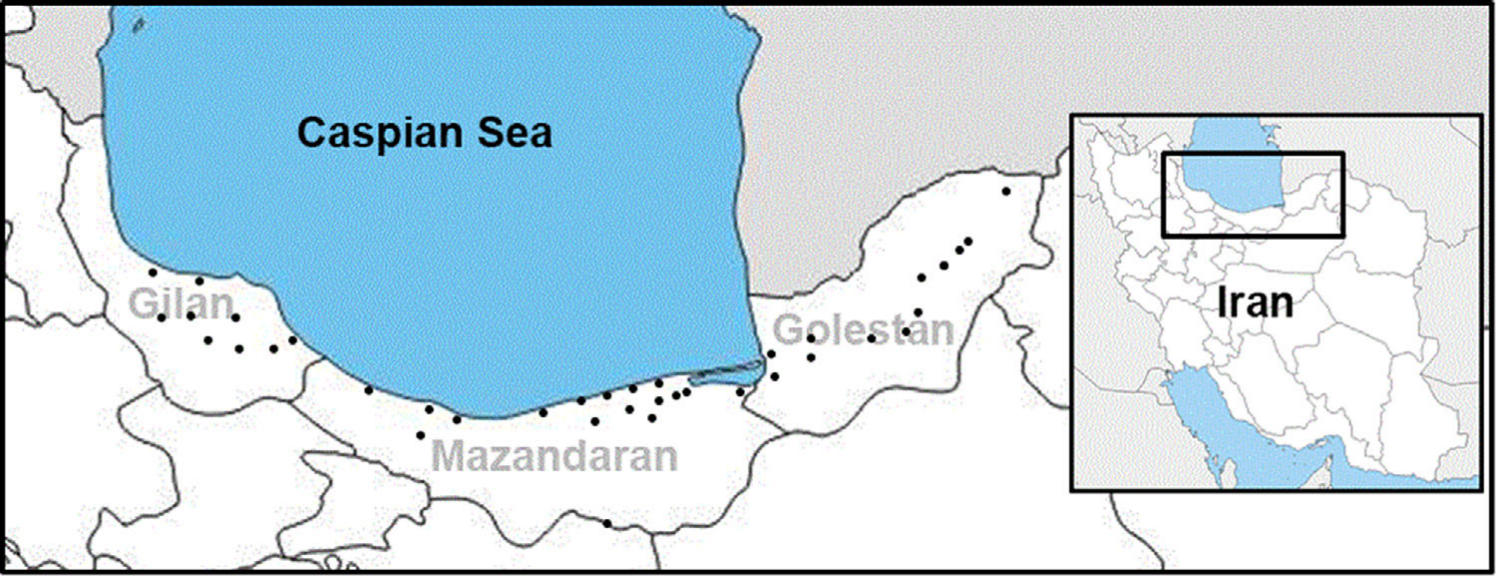
Geographical map of the swab collection locations of the current study

962 cloacal swabs were collected from domestic ducks being sold at different LPMs of the northern provinces of Iran (namely Golestan, Mazandaran and Gilan located along the southern coast of the Caspian Sea) in 2017 (Figure 1). The ducks had no apparent clinical signs. The swabs were placed in 2–3.5 ml of sterile phosphate‐buffered saline (PBS) with pH 7.0–7.4 that contained 0.1% antibiotic according to standard protocol (OIE, 2021). All specimens were kept at 4˚C and transported to the Avian Influenza Reference Laboratory within 24 h.

### 2.2 Virus isolation

Isolation procedure of the virus was done according to OIE guidelines (OIE, 2021). Briefly, the swab supernatant were centrifuged at 4000 × *g* for 5 min and then the supernatants were used to inoculate five 9‐ to 11‐day‐old specific pathogen free (SPF) embryonated chicken eggs of via the allantoic sac. The eggs were incubated at 37°C for 2–5 days. The allantoic fluid was collected from embryos that died post‐inoculation and chilled them at 4°C. It is worth mentioning that the eggs with dead embryos within 24 h post‐inoculation were discarded as non‐specific (Spackman, 2016).

### 2.3 Serological tests

The allantoic fluid was examined using haemagglutination (HA), haemagglutination inhibition (HI) and neuraminidase inhibition (NI) assays according to standard protocol (OIE, 2021). Briefly, for the HI and NI assays, specific antisera against H1–H16 and N1–N9 were used (OIE, 2021). The positive samples were subjected to the molecular assays for characterisation and phylogenetic analysis.

### 2.4 RNA extraction and RT‐PCR

RNA was extracted from HI‐ and NI‐confirmed allantoic fluid using High Pure Viral RNA Isolation kit (Roche, Germany). A preliminary one‐step RT‐PCR using H3‐specific primers (forward primer: 5′‐CARATTGARGTGACHAATGC‐3′ and reverse primer: 5′‐GGTGCATCTGAYCTCATTA‐3′) was performed to confirm the subtype as described previously (Lee et al., 2001). Next, each viral genome segment was amplified using universal primers previously described (Hoffmann et al., 2001). The products with correct sizes were gel purified and Sanger sequenced using the same primers.

### 2.5 Phylogenetic analysis

Datasets were prepared by using databases such as NCBI and GISAID. Additional isolates with highest BLAST homology were also added. This was carried out for each sequenced segment. A maximum likelihood tree was constructed using MEGA 7 software (Kumar et al., 2016) based on the general time‐reversible model of nucleotide substitutions (GTR+G) and bootstrap replicates of 1000. Evolutionary distances were inferred utilising maximum composite likelihood model, with rate variation among sites that was modelled with a gamma distribution (shape parameter = 1).

## 3 RESULTS

### 3.1 The duck‐derived isolates belonged to AIV H3 subtype

Of the 962 cloacal swabs, only five samples were positive for influenza virus as confirmed by HA assay and RT‐qPCR amplification. Three of the five isolates were identified as H3 subtypes based on HI assay and RT‐qPCR using H3‐specific primers. RT‐qPCR using the H5 and H9 specific primers came back negative (data not shown). Furthermore, all the three isolates were identified as N2 subtype in NI assay, and later confirmed by sequencing. In fact, the complete genome of the three H3 isolates (namely A/domestic duck/Iran/340/2017(H3N2), A/domestic duck/Iran/375/2017(H3N2), A/domestic duck/Iran/379/2017(H3N2)340, 375 and 379) were Sanger sequenced and deposited to GenBank (accession numbers shown in Table 1).

**TABLE 1:** Information of the Iranian avian influenza viruses of the current study

Isolate	Bird	Province	Date	Segment	Accession #
340	Domestic duck	Mazandran	2017/8/30	PB2	MW407061
				PB1 and PB1‐F2	MW407065
				HA	MW406901
				NA	MW406904
				PA and PA‐X	MW407048
				NP	MW406925
				M2 and M1	MW406905
				NEP and NS1	MW406926
375	Domestic duck	Mazandran	2017/8/9	PB1 and PB1‐F2	MW422783
				PB2	MW422785
				HA	MW422771
				NA	MW422775
				PA and PA‐X	MW422784
				NP	MW422780
				M2 and M1	MW422781
				NEP and NS1	MW422782
379	Domestic duck	Mazandran	2017/8/8	PB1 and PB1‐F2	MW422893
				PB2	MW422891
				HA	MW422885
				NA	MW422888
				PA and PA‐X	MW422890
				NP	MW422887
				M2 and M1	MW422886
				NEP and NS1	MW422895

### 3.2 The isolates belonged to the Eurasian lineage

**FIGURE 2:**
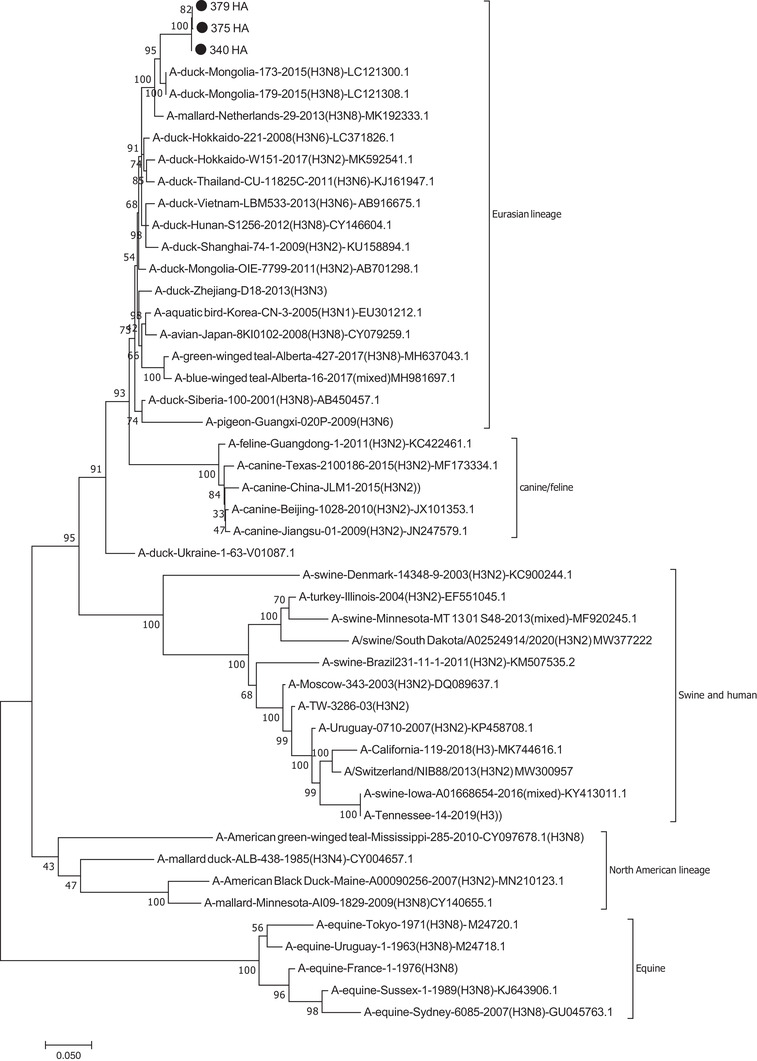
Phylogenetic tree of HA. The isolates of the current study clustered to a group including mostly AIV H3N8 subtype belonging to Eurasian lineage. The isolates are indicated with black circle

The HA genes of the isolates were almost identical at a nucleotide distance of <0.1% (Supplementary Table S1). When BLAST was run on the HA nucleotide sequences, the top homolog isolates (as of January 2021) were some H3N8 isolates from North Kazakhstan (97.1% to MN945300 and MN945304, also see Table 2). The rest of the matching sequences belonged to the duck and other aquatic birds isolated from East Asia and Europe, better known as the Eurasian lineage. Phylogenetic tree was also constructed and the results showed that the isolates of this study formed a cluster with isolates mostly belonging to H3N8 subtype (Figure 2). According to the tree, which was divided into American and Eurasian lineages, the H3 isolates of the current study clustered to the avian‐Eurasion lineage, but were distinct from canine, feline, equine, swine, human or turkey influenza viruses. They also clearly differed from the North American lineage. Furthermore, the isolates showed 91% nucleotide similarity to Dk/Ukraine/1/63(H3N8), one of the earliest known avian H3 strains (Bean et al., 1992). The isolates showed distances of ∼13%, 24%–30%, 27% and >34% with canine/feline, human, swine and equine isolates, respectively (Supplementary Table S1).

**TABLE 2:** List of viruses with the highest BLAST identity to each segment of the isolates of the current study as of January 2021

Segment	Gene(s)	Highest homolog influenza virus	GenBank accession number	Percentage of homology
1	PB2	A/mallard duck/Netherlands/35/2015(H4N6)	MF693922	97.03%
2	PB1 and PB1‐F2	A/mallard duck/Georgia/10/2016(H7N7)	MF694021	97.8%
3	PA and PA‐X	A/duck/Bangladesh/37203/2019(H7N1)	MT090472	97.38%
4	HA	A/garganey/North‐Kazakhstan/45/2018(H3N8)	MN945300	97.18%
5	NP	A/teal/Egypt/MB‐D‐621C/2016(H7N9)	MN208045	98.9%
6	NA	A/greater white‐fronted goose/Netherlands/3/2011(H6N2)	KX978364	96.95%
7	M2 and M1	A/pintail/Egypt/MB‐D‐384C/2015(H3N6)	MN208008	98.9%
8	NEP and NS1	A/goose/Karachi/NARC‐13N‐969/2014(H14N3)	KX602672	98.71%

*Note*: The isolates shared the same homologs.

According to our protein analysis, the three H3 isolates shared the same amino acid sequence (PEKQTR/GLF) at the cleavage site between the HA1 and HA2, further indicating that they belong to low pathogenic strains. Moreover, all the three H3 isolates shared the same N‐glycosylation sites at positions 24, 38, 54, 181, 301 and 499 aa. In addition, two amino acids of Q226 and T228 at the receptor binding site were identified, suggesting that the isolates bind to a‐2,3‐linked sialic acid receptors, which are generally recognised as the main receptors in avian species (Matrosovich et al., 2000; Wiley & Skehel, 1987).

**FIGURE 3:**
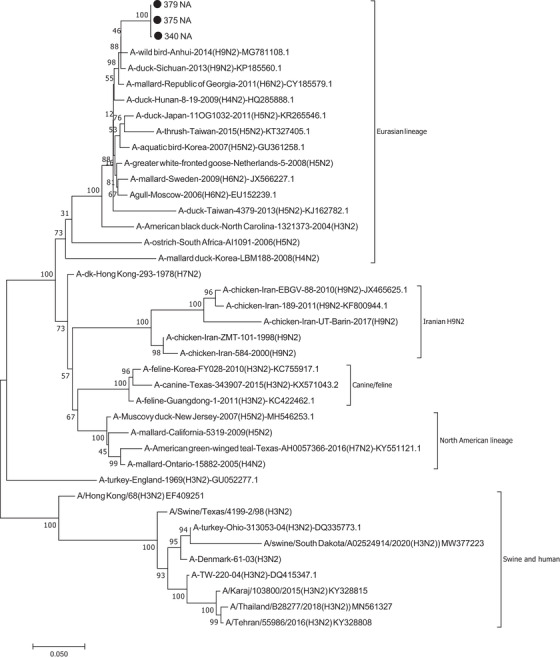
Phylogenetic tree of NA of the isolates of the currents study showed that they belong to the Eurasian lineage. The isolates are indicated with black circle

Similar analyses were performed for the segment 6 sequences. The NA genes of the three isolates were almost identical as their nucleotide distance was <0.2% (Supplementary Table S2). Furthermore, BLAST analyses showed highest homologies (max 96.5%–96.9%) to a variety of aquatic bird‐derived subtypes including H6N2, H4N2, H9N2, H1N2 and even H5N2, suggesting that the NA of the isolates belonged to N2 subtype. Figure 3 shows the location of the NA genes of the current study as compared to other NA selected as described in materials and methods. All the three isolates clustered to the avian‐Eurasian lineage. As seen in the tree, the isolates did not cluster to canine, feline, swine and human influenza viruses. Interestingly, there was only 74%–82% nucleotide similarity between the isolates of this study and chicken‐derived H9N2 strains previously reported from Iran (Supplementary Table S2). BLAST analysis also did not show any Iranian isolate in the top results.

### 3.3 Phylogenetic analysis showed all the internal protein genes were of avian origin

BLAST and phylogenic trees of the internal genes showed that the three isolates of this study belonged to the Eurasian lineage derived from aquatic birds (Supplementary Table S1–S8). For instance, BLAST analysis on the M gene (segment 7 including matrix M1 and M2 protein genes) showed that the three isolates had the highest nucleotide sequence homology with aquatic bird‐origin influenza viruses of different combinations such as H3N6, H10N7, H3N8, H7N7 and H11N8 (at most 98.7%, see Table 2). The M segment also showed 12% distances with those of human H3N2 isolates from Iran as well as 9%–14% distance with Iranian chicken H9N2 (Supplementary Table S3). As for the NP gene (segment 5), there was high homology )98.9%) to A/teal/Egypt/2016(H7N3) and A/teal/Egypt/2016(H7N9) isolates. However, other combinations such as H4N6 and H10N4 and H3N6, all isolated from aquatic birds, were among the next matching sequences (98.5%–98.7%). The nucleotide differences are available as Supplementary Table S4. Also analyses on the NS gene (segment 8 containing nuclear export protein (NEP) and non‐structural protein (NS1) genes) demonstrated that the isolates of this study were closely related to A/goose/Karachi/NARC‐13N‐969/2014(H14N3) and A‐mallard duck‐Netherlands‐2009(H5N3) (98.71%). Supplementary Table S5 also shows the nucleotide distances compared with NS of several strains isolated from other vertebrate species including swine, canine and Homo sapiens. Similar to the other segments, the distance to homo sapiens‐derived isolates were 16%–18%.

Analyses were also run on the viral polymerase protein genes (PA, PB1 and PB2); PA gene (segment 3, including PA and PA‐X genes) had the highest nucleotide sequence homology (97.38%) with to A/duck/Bangladesh/37203/2019(H7N1) followed by different subtype combinations (H4N6, H7N6, H2N2 etc.). The nucleotide differences are also available as Supplementary Table S6. In addition, Isolates A/mallard duck/Netherlands/35/2015(H4N6) and A/tufted duck/Georgia/1/2012(H2N3) were the closest homologs to the PB2 gene (segment 1) of isolates of this study, both matching at 97.12%. Supplementary Table S7 shows the nucleotide distance of PB2 compared to other strains, including those isolated from other vertebrates. The scores were distinct to those of Homo sapiens, swine and other vertebrates. The amino acids E627, D701 and S714, which are known to play crucial role in interspecies transmission (Czudai‐Matwich et al., 2014; Manz et al., 2013; Subbarao et al., 1993), were also present on all the three PB2 proteins. Furthermore, the PB1 gene (segment 2 including PB1 and PB1‐F2) of all the three isolates had the highest homology to A/mallard duck/Georgia/10/2016(H7N7) and A/Garganey/Bangladesh/38920/2019(H7N4) at 97.8% (lowest nucleotide distance 1.95%–2.14%, Supplementary Table S8).

Based on our findings, it was difficult to identify a particular geographical region, or a highly homologous isolate, as the original source of the AIV of this study. This was mainly because each genome segment showed homology to different strains (Table 2). Surprisingly however, no previously reported Iranian isolates showed up among the highly homologues BLAST isolates (>90%–95%), suggesting that the isolates of this study are the first of their kind from Iran.

## 4 DISCUSSION

The southern coast of the Caspian Sea is entirely within the Iranian territories. This region is divided into three provinces and is a common stopover site for migratory birds. In addition, due to the climate and abundance of lakes and wetlands, this region is also a natural habitat for resident waterfowls. Furthermore, these provinces, especially Mazandaran, are among the provinces with the highest number of poultry farms in the country. They also have an abundance of LPMs. However, such markets are of public health concern as they provide an environment for different animals and humans to come into close contact, thus increase the risk of infection with zoonotic viruses. Yet, these markets are a great source for identifying novel viruses and reassrortant AIV, thus should be continuously monitored. On the other hand, the Anatidae family, including both the free‐flying and domestic ducks marketed in LPMs, has been widely studied due to their potential in interspecies transmission of influenza (Fang et al., 1981; Guan et al., 2019; Li et al., 2016). Therefore, in this study we aimed to study this group of birds in LPMs of the northern provinces of Iran.

The H3 subtype is an important group of influenza viruses. Although the characterisation and antigenicity of H3 subtype influenza viruses vary in different host species, they pose public and poultry health risk due to the possibility of interspecies transmission. Furthermore, while the H3 AIV show no major clinical signs in aquatic birds, they may serve as donor for the exchange of genome segments with other subtypes such as H7 (Gao et al., 2013). Therefore, they may cross the species barrier and infect other animals, and therefore, they are of particular importance.

Several reports on the AIV of low (Fereidouni et al., 2010; Ghalyanchi Langeroudi et al., 2013; Heydarchi et al., 2010; Mehrabadi et al., 2018; Nili & Asasi, 2003) and high pathogenicity (Ghafouri et al., 2019; Yegani et al., 2019) have been published from Iran. However, most of the works have studied the poultry, and therefore, little is known about the status and current subtypes of the AIV circulating in the Iranian domestic and wild waterfowls. More than a decade ago, Fereidouni and colleagues detected H3 subtypes from aquatic birds, including a H3N8 from mallard (Fereidouni et al., 2010). Detection of H3 AIV from captive waterfowls in Tehran Zoo has been also reported (Heydarchi et al., 2010). However, in the current study we reported for the first time the isolation, whole genome sequence and characterisation of duck‐derived H3N2 AIV from Iran. All the genome segments clustered to the Eurasian lineage, although the highest homologs to each segment were different from the others (Table 2). The nucleotide distance and phylogenetic trees also showed that the isolates of the current study clustered to groups distinct from non‐bird vertebrates (Figures 2 and 3 and Supplementary Tables S1–S8).

Studies on the 1980s swine influenza isolates from Southeast Asia have shown that their HA genes are closely related to the AIV isolated from wild ducks (Pensaert et al., 1981). Furthermore, it has been shown that the AIV A/Duck/Ukraine/63 has served as progenitor of the H3 of human 1968 Hong Kong influenza (Fang et al., 1981). In the current study, phylogenetic analysis of the HA gene indicated almost 90% nucleotide similarity to the A/Duck/Ukraine/63 virus (Supplementary Table S1). Thus the HA genes of the isolates of the current study have undergone nearly 10% change in more than 50 years.

Furthermore, the high similarity of some of the internal protein genes, namely NP, PA and PB1 of the isolates of this study with those of H7 subtype was interesting (Table 2). In fact, the highest value of similarity among all the genes belonged to the NP gene as it showed almost 99% similarity to the highly pathogenic H7N9 strains. This was the same for all the three isolates and could further indicate the potential of low pathogenic strains to turn into pandemic viruses.

Furthermore, the risk of interspecies transmission between avian and canine/feline groups is generally considerable (Song et al., 2008). In agreement, Figure 2 and Supplementary Table S2 showed that the canine/feline were the closest group of vertebrates studied in this article at nearly 87% similarity.

The results of this study emphasise the importance of surveillance and monitoring AIV in LPMs of different provinces of Iran. Such analyses allow us to investigate the evolution and possible reassortment between different subtypes as well as spotting the emergence of viruses with pandemic potential.

## CONFLICT OF INTEREST

The authors declare that they have no competing interests.

## ETHICS STATEMENT

Animal handling procedures were performed in line with the national animal welfare regulations. The Institutional Animal Care and Use Committee (IACUC), Razi Vaccine and Serum Research Institute approved all animal experiments.

## AUTHOR CONTRIBUTIONS

AA performed the experiments and wrote the manuscript. AS, SAP, HP and MHFM revised the manuscript and supervised the study.

## CONSENT FOR PUBLICATION

All authors gave their consent for research publication.

## CONSENT TO PARTICIPATE

All authors contributed to the study conception and design. All authors read and approved the final manuscript.

### PEER REVIEW

The peer review history for this article is available at https://publons.com/publon/10.1002/vms3.819

## Supporting information

Supporting information

Supporting information

Supporting information

Supporting information

Supporting information

Supporting information

Supporting information

Supporting information

## Data Availability

Data sharing not applicable – no new data generated, or the article describes entirely theoretical research.
