# Supplementary material for: Characterisation, whole‐genome sequencing and phylogenetic analysis of three H3N2 avian influenza viruses isolated from domestic ducks at live poultry markets of Iran, 2017: First report
Source: Vet Med Sci. 2022 Jun 2;8(4):1594–602. doi: 10.1002/vms3.819 (PMC9297799; doi:10.1002/vms3.819)
Supplement: Supplementary file 6 — Supporting information [file VMS3-8-1594-s006.docx]

| Gene Bank sequences  This study sequences | **A-duck-Bangladesh-2018(H4N6)-MN209302.1** | **A-duck-Bangladesh-38292-2019(H2N2)** | **A-domestic_green-winged_teal-Hunan-2006(H5N1_-KC690155.1** | **A-duck-Bangladesh-2018(H7N6)-MN209366.1** | **A-turkey-2004(H3N2)-EF551044.1** | **A-chicken-Iran-2006(H9N2)-JQ994241.1** | **A-Mallard-Netherlands-2013(H3N6)-MK414739.1** | **A-swine-North_Carolina-2003(H3N2)-EF551052.1** | **A-avian-Canada-1994(H7N1)-MH558687.1** | **A-canine-Beijing-362-2009(H3N2)** | **A-migratory_duck-Jiang_Xi-2004(H4N6)-EF597427.2** |
| --- | --- | --- | --- | --- | --- | --- | --- | --- | --- | --- | --- |
| **379 PA** | 0.029 | 0.014 | 0.039 | 0.030 | 0.133 | 0.091 | 0.038 | 0.120 | 0.067 | 0.067 | 0.035 |
| **340 PA** | 0.027 | 0.014 | 0.038 | 0.028 | 0.134 | 0.095 | 0.038 | 0.122 | 0.065 | 0.066 | 0.032 |
| **375 PA** | 0.029 | 0.015 | 0.039 | 0.030 | 0.133 | 0.091 | 0.038 | 0.120 | 0.067 | 0.067 | 0.035 |

Supplementary Table S6. Estimates of Evolutionary Divergence between PA Gene Sequences

The number of base substitutions per site from between sequences are shown. Analyses were conducted using the Maximum Composite Likelihood model. This analysis involved 55 nucleotide sequences but for easier presentation only 11 are showed in table above. There were a total of 1948 positions in the final dataset. Evolutionary analyses were conducted in MEGA X [1].

1. Kumar S., Stecher G., Li M., Knyaz C., and Tamura K. (2018). MEGA X: Molecular Evolutionary Genetics Analysis across computing platforms. Molecular Biology and Evolution 35:1547-1549.
